# Approaches to protocol standardization and data harmonization in the ECHO-wide cohort study

**DOI:** 10.1038/s41390-024-03039-0

**Published:** 2024-02-16

**Authors:** Lisa P. Jacobson, Corette B. Parker, David Cella, Daniel K. Mroczek, Barry M. Lester, on behalf of program collaborators for Environmental influences on Child Health Outcomes, P. B. Smith, K. L. Newby, D. J. Catellier, R. Gershon, D. Cella

**Affiliations:** 1 Department of Epidemiology, Johns Hopkins University Bloomberg School of Public Health, Baltimore, MD USA; 2 Research Triangle Institute, Research Triangle Park, NC USA; 3 Department of Medical Social Sciences, Northwestern University, Chicago, IL USA; 4 Department of Psychology, Northwestern University, Evanston, IL USA; 5 Department of Psychiatry & Human Behavior, Alpert Medical School of Brown University, Providence, RI USA; 6 Department of Pediatrics, Alpert Medical School of Brown University, Providence, RI USA; 7 Coordinating Center: Duke Clinical Research Institute, Durham, NC USA; 8 Research Triangle Institute, Durham, NC USA; 9 Person-Reported Outcomes Core: Northwestern University, Evanston, IL USA

## Abstract

**Abstract:**

The United States (U.S.) National Institutes of Health–funded Environmental influences on Child Health Outcomes (ECHO)-wide Cohort was established to conduct high impact, transdisciplinary science to improve child health and development. The cohort is a collaborative research design in which both extant and new data are contributed by over 57,000 children across 69 cohorts. In this review article, we focus on two key challenging issues in the ECHO-wide Cohort: data collection standardization and data harmonization. Data standardization using a Common Data Model and derived analytical variables based on a team science approach should facilitate timely analyses and reduce errors due to data misuse. However, given the complexity of collaborative research designs, such as the ECHO-wide Cohort, dedicated time is needed for harmonization and derivation of analytic variables. These activities need to be done methodically and with transparency to enhance research reproducibility.

**Impact:**

- Many collaborative research studies require data harmonization either prior to analyses or in the analyses of compiled data.
- The Environmental influences on Child Health Outcomes (ECHO) Cohort pools extant data with new data collection from over 57,000 children in 69 cohorts to conduct high-impact, transdisciplinary science to improve child health and development, and to provide a national database and biorepository for use by the scientific community at-large.
- We describe the tools, systems, and approaches we employed to facilitate harmonized data for impactful analyses of child health outcomes.

## Introduction

The landmark Environmental influences on Child Health Outcomes (ECHO; https://www.nih.gov/echo) research program was launched in 2016 by the National Institutes of Health (NIH).^1–3^ The ECHO Program includes the ECHO-wide Cohort Study (EWC), an observational cohort created by pooling existing studies, and the Institutional Development Award (IDeA) States Pediatric Clinical Trials Network (ISPCTN) that centers on intervention research among children from 17 states generally underrepresented in clinical trials. The EWC was established to conduct high impact, transdisciplinary science to improve child health and development and to provide a national database and biorepository for use by the scientific community at-large. In this review article, we focus on two key challenging issues in the EWC: data collection standardization and data harmonization.

The EWC was established to address issues that may not be addressed by individual studies, which typically focus on a single outcome area or exposure, have limited statistical power to study rare clinical outcomes, and have limited generalizability. Pooling cohorts with common outcomes of interest and harmonizing data enable the EWC to conduct in-depth analyses of critical issues and account for confounding and modification unencumbered by traditional limitations, including sample size, diversity, and individual cohort characteristics.

The EWC began with existing pregnancy, birth, and early childhood cohorts that were collecting longitudinal data and expanded recruitment and continued follow-up. A total of 69 cohorts funded by 31 awards were involved in contributing data from >57,000 children from diverse backgrounds across the United States (U.S.). Five outcome areas were targeted: (1) pre-, peri-, and postnatal outcomes; (2) upper and lower airway conditions; (3) obesity; (4) neurodevelopment; and (5) positive health.

In ECHO, environmental exposures include the totality of early life conditions, not just traditional exposures, such as air pollution and chemical toxicants, but also home, neighborhood, socioeconomic, behavioral, and psychosocial factors. ECHO chose these factors because they pinpoint modifiable aspects of the environment. ECHO’s goal is to inform programs, policies, and practices by illuminating the risk factors of poor child outcomes and protective factors that buffer the child and facilitate resilience.

The 69 cohorts represent a demographically diverse cross-section of U.S. geographic regions, including children from metropolitan and rural populations and with socioeconomic heterogeneity. The EWC includes cohorts from Native American populations and cohorts with over-representation of Black/African American and Hispanic/Latino children. ECHO, comprising the EWC and ISPCTN, promotes translating observational research into intervention trials and accelerating the development of solution-oriented treatments. As of February 14, 2022, ECHO had 909 published articles (https://echochildren.org/echo-program-publications/).

The EWC database comprises extant data collected by the cohorts prior to ECHO and new data collected by the cohorts using a common protocol. The combined data provide a powerful resource for the pediatric research community. Leveraging the existing infrastructure and extant data, EWC investigators developed and implemented the ECHO-wide Cohort Protocol (EWCP) to launch this large-scale collaborative research study.^2,4,5^

Successful EWC science requires both *standardization* of new data collection and *harmonization* of the extant data containing different measures used by the cohorts to assess data elements or constructs of interest. The EWCP defines the data elements for new data collection and extant data transfer; data elements are deemed either essential (must collect) or recommended (collect if possible) for new data collection. These designated data elements also are the required set to be submitted by cohort investigators from their existing data. In addition, cohorts have other related extant data to be submitted for potential harmonization. The EWCP further specifies preferred and acceptable measures that cohorts may use for new data collection. The use of multiple measures for the same element requires harmonization to capitalize on the breadth of data offered by the EWC. Here, we describe harmonization practices that advance collaborative research studies to answer compelling questions about pediatric health.

## The ECHO-wide Cohort approach

As described by LeWinn and colleagues,^2^ the EWC Protocol Working Group developed the EWCP to standardize new data collection. They describe the Protocol Working Group life stage subcommittees, interactions with Outcome Working Groups and the Alternative Measures Task Force, and the processes for managing communication across these constituents.

In this review article, we demonstrate how the EWC collaborative structure was further mobilized to achieve large-scale, high impact science, including novel approaches for data harmonization. We provide details on the Data Analysis Center (DAC) Data Systems used by the cohorts to map and upload data, efforts by the Person-Reported Outcomes (PRO) Core to link and otherwise harmonize similar measures of latent constructs, and the processes overseen by the Data Harmonization Working Group (DHWG) that support data harmonization as prioritized by Steering Committee-approved analysis proposals.

The cohorts that contribute to the EWC are heterogeneous in participant demographics, enrollment criteria, follow-up period, data elements, data collection modes, and study designs. Whereas combining data from these cohorts improves the generalizability and transportability of research findings, creating a Common Data Model (CDM) with such content diversity is a great challenge. By limiting the data collection measures, a common protocol standardizes new data collection. However, incorporating existing data into the CDM requires extensive harmonization. The DHWG was therefore established to coordinate harmonization efforts and to develop best practice guidelines. Harmonization is the responsibility of all components, including the DAC, the PRO Core, and substantive experts from various cohorts. Here, we describe harmonization challenges and our approaches to facilitate analyses using a CDM.

## Standardizing new data collection

As previously described,^2^ the EWCP defines the elements that constitute the EWC platform for analysis and the measures that cohorts should use for new data collection. These elements are listed according to participant life stage: prenatal, perinatal, infancy, early childhood, middle childhood, and adolescence.

An initial decision surrounded whether the study would require cohorts to use the same measures to collect data during the life stage or whether cohort-specific measures would be allowed. Using the same measure increases standardization and facilitates a quicker and less error-prone path to data analysis. Using cohort-specific measures allows implementation with less new training and facilitates longitudinal analyses of legacy measures within a cohort but ultimately requires harmonization prior to analysis of data across cohorts. For each essential data element, the protocol allows cohorts to use preferred or acceptable measures for its collection, with the understanding that the data may be harmonized. In some instances, cohorts were allowed to continue to collect data with measures that they previously used; these legacy measures were defined as “alternative” measures. An Alternative Measures Task Force developed the process for investigators to use when requesting the inclusion of an alternative measure in subsequent versions of the protocol. This was the first step toward ECHO-wide standardization and data harmonization.

In addition to the essential core elements that all cohorts are required to collect from participants during a specific life stage, the protocol also contains recommended elements. These elements provide data for a deeper investigation into an area. Not all cohorts need to collect data for a recommended element, but if so desired, the measure listed on the protocol should be used for new data collection.

Measures delineated in the protocol include proprietary instruments, other standardized and validated instruments, data collection forms modified from the cohorts, and new instruments.

**Fig. 1:**
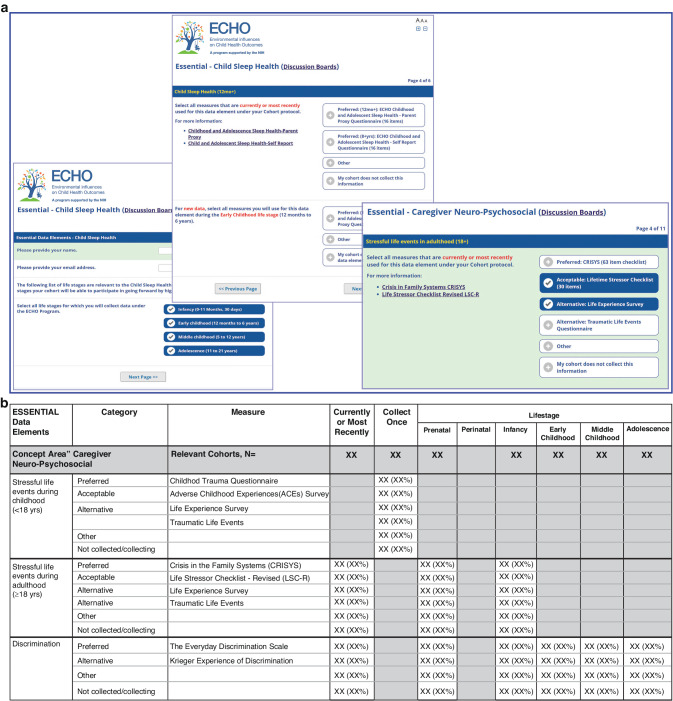
Demonstration of how the Cohort Measurement Identification Tool was used for collecting and reporting metadata about planned data collection. **a** shows, in select screenshots, how the cohort used the tool to identify the relevant life stage, select the measures that will be used to collect data on Child Sleep Health (an outcome), and on Caregiver Stressful Life Events (an exposures). **b** is an excerpt from the Cohort Measurement Identification Tool summary report template that demonstrates how the Caregiver Stressful Life Events measures selected by the cohorts were summarized by frequencies across life stages.

As part of the protocol development process, the DAC developed the Cohort Measurement Identification Tool (CMIT). For every element in each life stage, each cohort was asked to identify the measure(s) they most recently used and which proposed protocol measure they planned to use for new data collection; Fig. 1a shows the CMIT survey instrument with pages for identifying the relevant life stage (left Panel), the collection of information on the Sleep Health outcome in the relevant life stage (middle Panel) and Stressful Life Events, one of the many potential exposures, in its relevant life stage (right Panel). Figure 1b shows an excerpt from a report template summarizing the Stressful Life Events across cohorts.

**Fig. 2:**
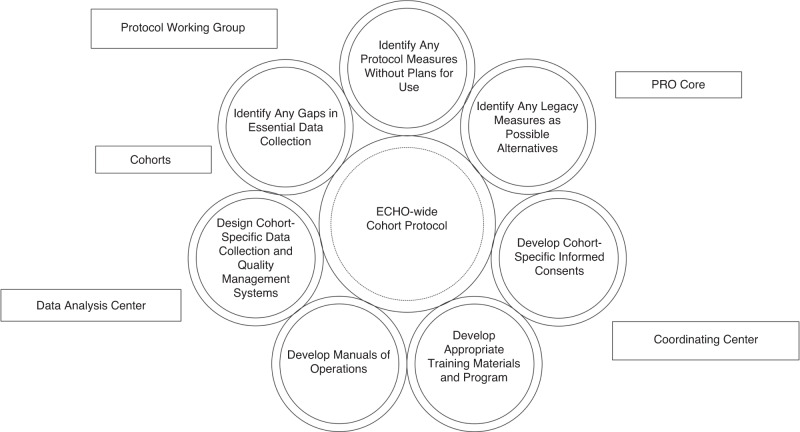
Collaborative uses of the Cohort Measurement Identification Tool (CMIT).

Using this information, the Protocol Working Group revised the protocol draft to delete measures that were rarely selected (i.e., measures the cohorts did not plan to use). The responses also identified legacy measures used by multiple cohorts for the Protocol Working Group to consider for inclusion as preferred, acceptable, or alternative measures. Lastly, the responses helped ECHO components to understand the complexities across the cohorts and to prepare for implementation. Figure 2 highlights varied uses of the CMIT tool to evaluate the draft protocol and to begin the development of necessary materials and systems for implementation.

## Data systems

**Fig. 3:**
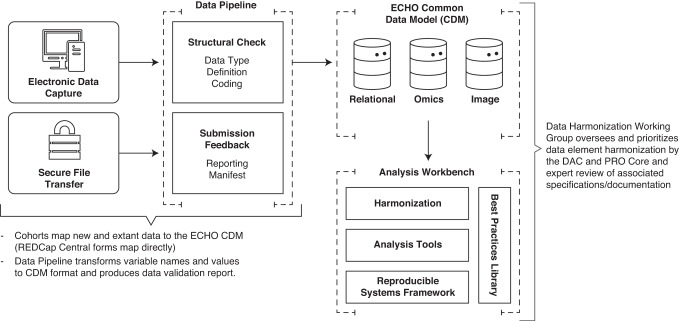
Data systems managed by the Data Analysis Center (DAC).

The DAC developed highly customized, web-based systems and tools to register cohort participants and classify them according to the extent of their participation in the EWC (e.g., contributing new and extant data versus contributing only extant data); transform data collected in local systems to a format consistent with the CDM; track biospecimen collection, processing, and storage; and capture new data (Fig. 3). A tool branded Data Transform allows cohorts to provide all the necessary details (the “roadmap”) to the DAC for converting existing and new data from cohort data systems to the EWC structured-query-language (SQL) server database. The data capture system, based on Research Electronic Data Capture (REDCap)^6,7^ and named REDCap Central, allows cohorts to directly administer and enter data collected from participants in a secured web-based system. Cohorts can use REDCap Central, a local data capture system, or a hybrid of the two for new data collection (Fig. 3). Cohorts using a local data capture system map and transfer new data similarly to extant data.

For new data, each cohort selects its planned structure of visits and protocol measures within appropriate life stages from Data Transform menus. If using REDCap Central, the selected visits and measures within life stages then become visible in the REDCap Central dashboard with the participants loaded from the registration system. In addition to cohorts utilizing REDCap Central for interactive data entry, the DAC developed a survey manager for cohorts to send surveys directly to participants via e-mail. The survey allows cohorts to send a single e-mail for multiple surveys and guides the participant via a custom menu indicating surveys that are completed and surveys remaining to be completed. This advanced remote administration of the measures has become critically important during the period of social isolation due to the COVID-19 pandemic. Another feature that enhances accessibility and utilization of the REDCap Central data capture system is a multilingual support module within REDCap Central that allows cohorts and ECHO participants to toggle between English and Spanish versions of the data collection forms.

## Extant data

Since cohorts collected data prior to ECHO, the DAC initially focused on the development of systems to transform and load disparate data to the CDM. These systems and related processes supported early harmonization efforts. Since cohorts would have the best knowledge about their extant data and how they relate to the CDM, the cohorts used Data Transform to: (1) confirm or modify the visit structures that were initially identified using the CMIT tool; (2) select the forms and measures for which they collected data elements in the protocol, either exactly as stated in the CDM or related data for the elements of interest; (3) map their data formats to the formats specified in the CDM data dictionaries or to customized data dictionaries, which the cohorts created based on standardized formatting that would be expected in pipeline processing; and (4) upload their data accordingly.

Prior to the development of the protocol, the DAC administered surveys to the cohorts to gather information about their individual cohort studies and populations, and subsequently placed this information in a metadata catalog. These surveys, administered in modules, ascertained information about the type of data that existed in each cohort (i.e., the domains) according to life stage. The metadata catalog permits faceted browsing and contains advanced search features that facilitate interactive searching and summarization of the metadata by investigators.

On the CMIT survey, the cohorts reported instruments in current use, including any protocol-named measures. The DAC used this information and information that the PRO Core gathered in interviews with some of the cohorts to develop a tool that listed all the forms on the protocol and more than 400 related forms. Cohorts were asked to indicate the forms for which they had existing data. For those forms reported by more than two cohorts, the DAC and PRO Core developed data dictionaries if the form was a standardized instrument. Otherwise, the cohorts submitted customized data dictionaries, which the DAC then reviewed to confirm that their formats adhered to that required by a data pipeline developed by the DAC for standard processing. As of April 2022, a total of 605 customized data dictionaries were in use by cohorts for submission of data. Each of these forms requires data harmonization, reflecting the magnitude of the harmonization effort.

## Data harmonization approaches

**Fig. 4:**
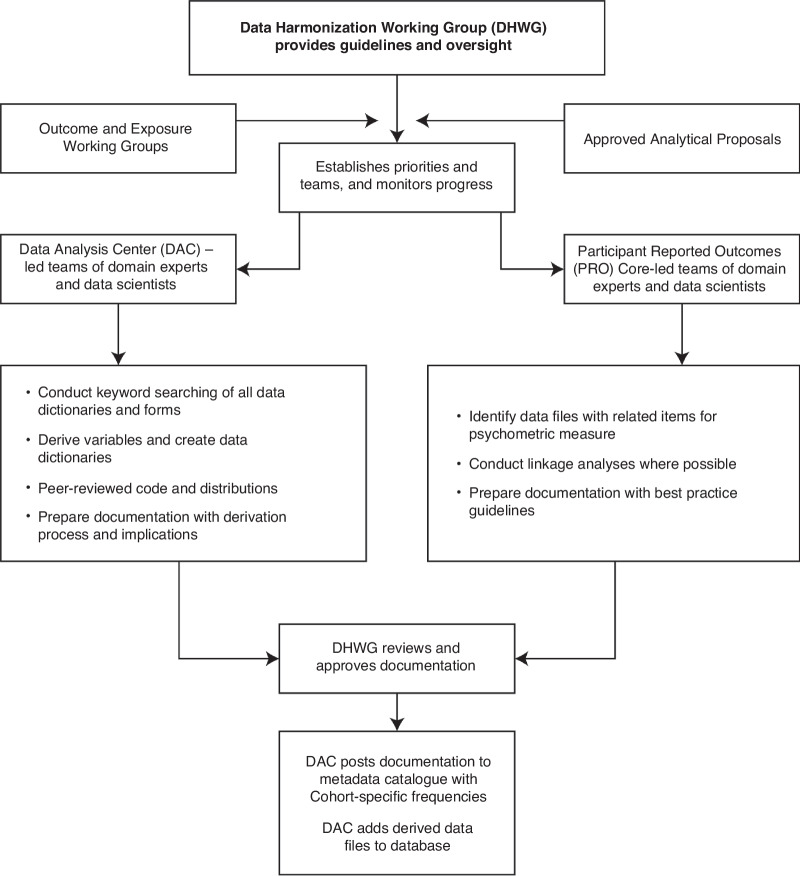
Central data harmonization processes following receipt of data files from the cohorts.

Many approaches exist for analyzing individual-level data collected with multiple measures. These include: (1) joint latent variable modeling of item-level data using item response theory (IRT), (2) harmonization through identification of commonalities and linkages *prior to* statistical analysis, (3) central review of cohort-specific data to identify common threads and instrument linking when possible (see indirect harmonization description). Fig. 4 shows how DAC and PRO Core review and harmonize data following their receipt. Other approaches take place during data analysis. Harmonization usually refers to measurement harmonization, which includes linking two or more measures of the same construct, such that a score obtained on one can be expressed as a score on the other(s). However, harmonization can also refer to alignment across studies of types of statistical estimators, functional form of models (e.g., linear vs. polynomial), or sets of predictors and covariates.^8^ It is important to keep in mind that there are many ways in which heterogeneity of study features and measures may introduce unwanted variation when attempting cross-study data synthesis. As such, most EWC data analyses require some degree of harmonization.

Harmonization at the level of measures can be classified as Direct methods of alignment. An example of a direct method is to transform or standardize scores under an invariant component by first ensuring factorial invariance for each unidimensional construct and using the invariant factor component to perform a transformation that allows some degree of comparability across different scores. Indirect methods make use of the finer distinctions in the data, namely item-level information. Methods for harmonizing item-level data include moderated nonlinear factor analysis^9^ and IRT alignment.^10^ Test-equating techniques, such as those used in PROsetta Stone (https://www.prosettastone.org/),^11–14^ may be used when the measures share common or overlapping items. Imputation of a missing variable based on prior information is another common approach to harmonization. However, the use of existing correlational data to impute values of missing information assumes that the high correlation observed in other sample(s) is present in the target sample. This assumption may not be the case and should ideally be checked in subsequent confirmatory work. Data harmonization may also require evaluation of heterogeneity across samples or cohorts (e.g., Cochran’s Q). When total scores or T-scores for different PROs can be harmonized to a common metric, analysts can convert scores to the common metric using crosswalk tables and perform individual-level analyses that include all participants with data on the harmonized measures (pooled analysis or mega-analysis^15,16^). While not as precise as item-level analyses, this approach is much simpler to implement and allows all harmonizable data to be used in a single individual-level analysis model.

### Indirect method example—harmonizing depression

Depression is a commonly assessed construct in ECHO. We first evaluated measures from multiple cohorts for common item overlap (i.e., the same question on different measures). With the advent of item banks beginning in the 1990s, increasing numbers of shared items can be found across different measures for the same construct and others. For example, stress and anxiety may share items with some depression measures. Linking functions, test-equating algorithms, and co-calibration all require sets of common items. Depression-related items from all the various measures can then be combined into a common dataset and an IRT model that includes a test-equating or linking function uses the overlapping items to first define a common depression metric and secondarily, estimate item location and discrimination parameters based on that underlying metric. Individual person-level scores can then be derived from whatever set of items a given person was administered. For example, using EWC data, a recent publication linked PROMIS® Depression with the Edinburgh Postnatal Depression Scale.^11^ Using a dataset in which both questionnaires were administered, the full set of items were calibrated onto a single, unidimensional construct (i.e., ‘depression’). This now allows one to “crosswalk” scores from one to the other, thereby harmonizing analyses where one or another of the questionnaires was used.

### Hierarchical example—harmonizing gestational age

Harmonizing some types of variables is more straightforward than harmonizing others. Gestational age can often be directly harmonized without use of complex linking approaches. Some bias, misclassification, and loss of precision will occur when combining estimates based on dating ultrasound scans, last menstrual period, or self-reported information. For gestational age, we include a hierarchy of parameters (Supplementary Table 1) used for the estimation and the various sources that were available for an individual. Therefore, analysts may conduct sensitivity analyses by data source to assess the impact on the results, such as when assessing the performance of a placental analyte in maternal serum for predicting an adverse pregnancy outcome.

### Simple comparable variable linkage example—harmonizing units or time

Differences in variable units, such as days to weeks, may be easily converted. For example, the number of cigarettes smoked per day can be converted with relative ease to cigarettes smoked per week, although some questions may elicit more precise and accurate counts than other questions. When similarly asked, pooling of data across studies can occur without much concern about measurement differences creating artifacts in results. Similarly with laboratory or analyte results, there are different mathematical algorithms used for standardization by urinary dilution^17^ and treating undetectable values for data which have been generated. When distributed approaches are used to generate new data, use of standards and reliability samples, such as blinded duplicates or common sample, are two commonly used approaches for quality assurance and adjustment.

### ECHO use of meta-analysis for harmonization

When pooled or mega-analysis^15,16^ is not possible due to a lack of harmonized measures, meta-analytic techniques may be used (i.e., conduct analyses in clusters of cohorts that used the same measure and then synthesize the results across the clusters). Prior to central availability of EWC data, the DAC provided statistical code to cohorts for implementation and meta-analyzed the results.^18,19^ This distributed collective analysis^5^ is also known as “coordinated analysis,”^20^ “parallel analysis,” or “coordinated meta-analysis.”^21,22^ This type of analysis differs from traditional meta-analysis based on published results since we controlled the statistical methods used by each cohort. With centrally available individual-level data, we may still stratify analyses based on clusters of cohorts and pool the resulting estimates using meta-analysis (e.g., weighted summaries of effect sizes) to synthesize findings while still preserving cohort and measurement heterogeneity. We and others also use additional approaches to address cohort heterogeneity.^23,24^

## Data harmonization working group (DHWG)

The enormity of the data elements in the EWCP, the large number of cohorts, and the related instruments that have been used over time necessitate parallel data harmonization processes in ECHO. The DHWG was responsible for developing EWC data harmonization guidelines to ensure a consistent approach across the program, and best practices for data harmonization, adhering to the principles of fairness, inclusiveness, and accuracy. Investigators from all ECHO components self-selected into this cross-cutting group.

The DHWG prioritized harmonization for: (1) common exposures, (2) primary child health outcomes, and (3) psychological and other latent variables constructed from instruments on the protocol. The DHWG initially asked Outcome and Exposure Working Groups to prioritize five constructs that they envisioned imminently needing and to name individuals from their groups to contribute to these harmonization efforts. The DHWG then established teams and provided templates for data harmonization processes and documentation.

Concurrently, the DAC initiated harmonization of variables needed for approved analyses, and the PRO Core started conducting linking analyses of psychometric measures. The DHWG integrated these lists with the variables prioritized for DHWG teams.

When cohorts could not directly map their data to the CDM, they submitted cohort-specific data files using custom data dictionaries. The DAC developed an R script that uses keywords to systematically search and report on form questions and responses found across all data dictionaries and SQL tables that potentially relate to the data construct. For example, to harmonize data on income, keywords included income, wage, and salary. We searched data tables and the dictionaries since related data may be found in text fields in the data tables and in variable descriptions in the data dictionaries.

Harmonization teams review the reports (variable descriptions, response categories, and data content/text) to determine relevance. For example, to study prenatal opioid use, the word “pain” identified pain medications but also picked up the environmental exposure ‘paint.’ Harmonization requires consideration of all potentially related data. The team reviews the source that contained the identified information since there may be related variables beyond those detected by the keyword search. After determining commonalities, the team derives analytical variables. We incorporate external data, such as reference tables required for normalization and standardization and note their sources in documentation files. Creating derived variables facilitates standardization across analyses. Transparent documentation that describes the process and deliberations facilitates decision-making by subsequent users of the data and reproducibility by other researchers.

## Checking the harmonization

Quality assurance of data harmonization assesses accuracy (see example below) and applicability (usefulness for end users). The teams creating the harmonization plans include experts in the subject matter who are familiar with the related body of literature so that the derived variables are of use to the greater scientific community. The DHWG reviews harmonization documents and places them in central locations for review by the ECHO community. The derivations, challenges, and resolutions are peered-reviewed by the DAC statistical team, and distributional properties of derived variables are provided in the documentation. A metadata catalog of the EWC database that is managed by the DAC contains the final harmonization documents, including frequencies of derived variables by cohort so that each cohort may review and confirm for accuracy.

When ECHO teams harmonize instruments that represent nesting or item reduction (e.g., a short form created from a long form), the correlation and scoring are checked by mimicking the reduction within the longer form data file. Graphically, the correlation is examined with scatterplots and the score agreement using Bland–Altman plots. This approach is demonstrated using the Wechsler Intelligence Scale^25,26^ in the Supplementary material.

## Conclusion

Collaborative study designs require data standardization and harmonization. In the EWC, standardization is evident in the creation of the data collection protocol with its manual of procedures, data collection forms, and policies for study practices, including data sharing, data harmonization, and publication. Data standardization using the CDM and derived variables should facilitate timely analyses and reduce errors due to data misuse. However, given the complexity of the EWC, dedicated time is needed for harmonization and derivation of analytic variables. These activities need to be conducted methodically and with transparency to enhance research reproducibility. Establishing a DHWG with membership from across the ECHO Program and having the group define, monitor, and document the data harmonization and standardization process, helps accomplish these goals.

### Supplementary information

Supplementary Figure

Supplementary Text

Supplementary Table 1

## Data Availability

A restricted version of the EWC data may be requested from the NICHD Data and Specimen Hub (https://dash.nichd.nih.gov/study/417122; 10.57982/ng1v-pz07).
