# Supplementary material for: Approaches to protocol standardization and data harmonization in the ECHO-wide cohort study
Source: Pediatr Res. 2024 Feb 16;95(7):1726–33. doi: 10.1038/s41390-024-03039-0 (PMC11245389; doi:10.1038/s41390-024-03039-0)

Supplemental Fig. 1a: Correlation between the constructed Wechsler Intelligence Scale for Children® Fifth Edition Verbal IQ (WISC5 VIQ) and the WISC 3rd Edition Verbal IQ (WISC3 VIQ)

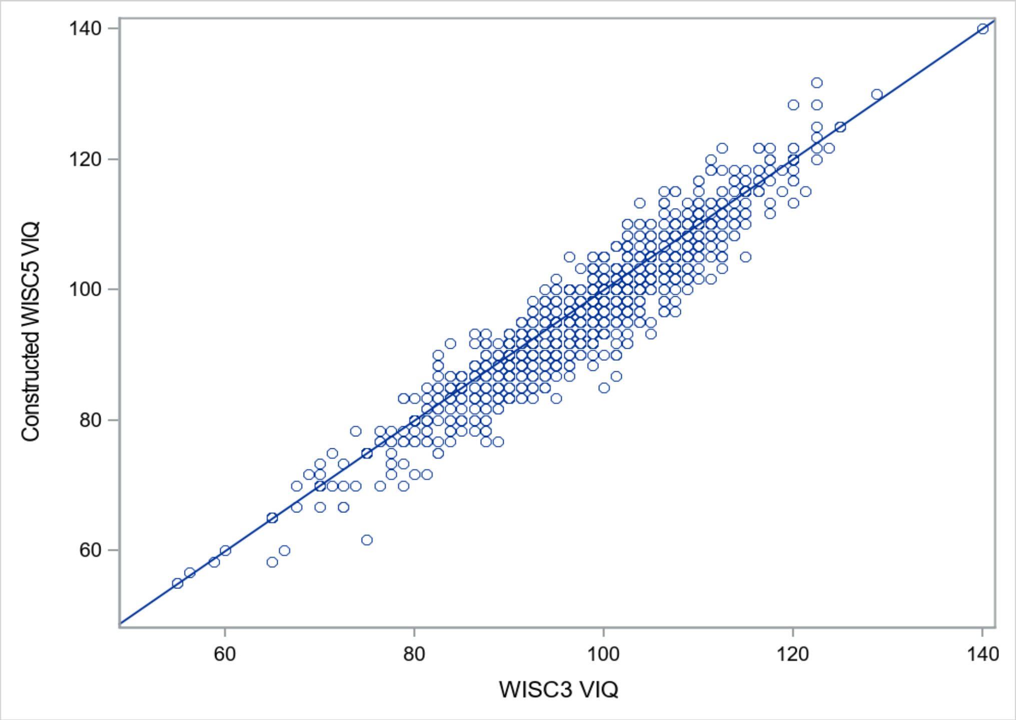

Supplemental Fig. 1b: Bland–Altman plot comparing the differences between the constructed Wechsler Intelligence Scale for Children® Fifth Edition Verbal IQ (WISC5 VIQ) and the WISC 3rd Edition VIQ (WISC3 VIQ) with the mean of the two scales

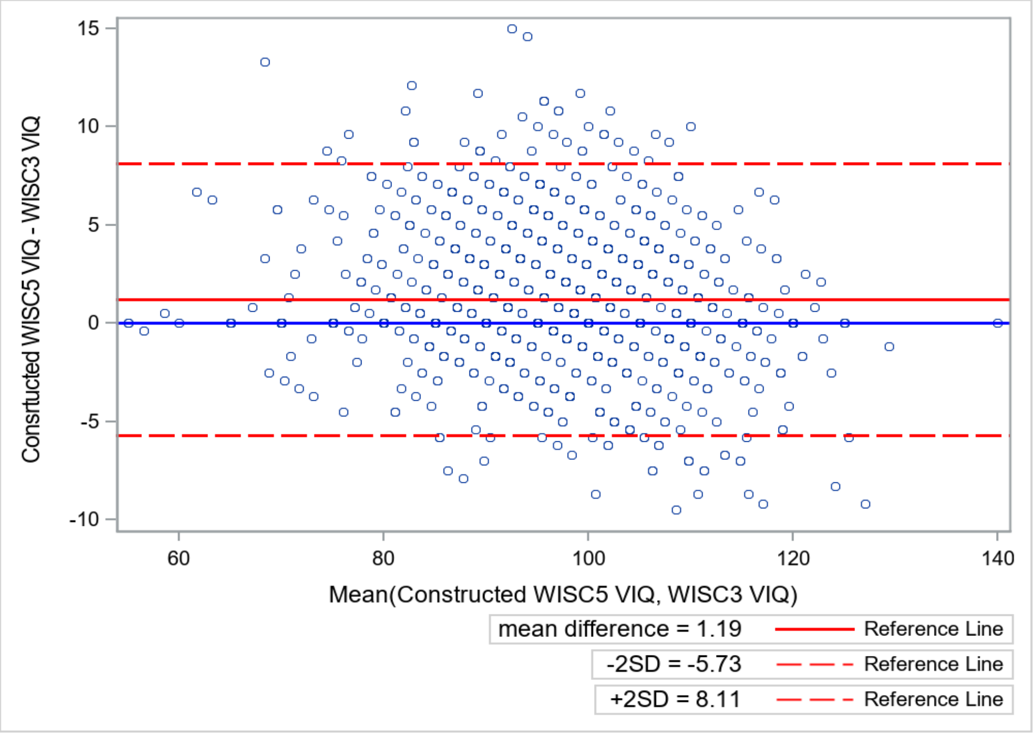

Supplement: Supplementary file 1 — Supplementary Figure [file 41390_2024_3039_MOESM1_ESM.pdf]
