# Supplementary material for: Approaches to protocol standardization and data harmonization in the ECHO-wide cohort study
Source: Pediatr Res. 2024 Feb 16;95(7):1726–33. doi: 10.1038/s41390-024-03039-0 (PMC11245389; doi:10.1038/s41390-024-03039-0)
Supplement: Supplementary file 2 — Supplementary Text [file 41390_2024_3039_MOESM2_ESM.pdf]

Example of Checking the Harmonization.

Here we use the Wechsler Intelligence Scale Children® Verbal IQ (WISC VIQ) to demonstrate how we check the correlation and scoring of nested scales in our harmonization process. In this situation, we constructed the WISC Fifth Edition (WISC5) VIQ scale[28] from the WISC Third Edition (WISC3) VIQ scale[29] by removing the Digit Span Scale Score and only using the Similarities Scale Score, Vocabulary Scale Score, Information Scale Score, and Comprehension Scale Score, which are common between the two versions.

Although the correlation was excellent (Supplemental Figure 1a), some bias was detected (Supplemental Figure 1b) (i.e., the mean difference [95% confidence interval] in this sample of 1400 was 1.19 [1.01,1.37]). We provide an indicator for which verbal IQ instrument was used in the harmonized data so that analyses may decide to accept the slight bias, restrict to one instrument, or perform sensitivity analyses.
