# Supplementary material for: Approaches to protocol standardization and data harmonization in the ECHO-wide cohort study
Source: Pediatr Res. 2024 Feb 16;95(7):1726–33. doi: 10.1038/s41390-024-03039-0 (PMC11245389; doi:10.1038/s41390-024-03039-0)
Supplement: Supplementary file 3 — Supplementary Table 1 [file 41390_2024_3039_MOESM3_ESM.pdf]

Supplemental Table 1. Hierarchy Used to Harmonize and Derive Gestational Age (GA) in ECHO-wide Cohort Study from Highest to Lowest Level of Use According to Information on Source

1= Dating based on embryo placement date following in vitro fertilization; or dating based on artificial insemination date (maternal medical record: Use of assisted reproduction 'yes' for this pregnancy and estimated date of delivery based on ART dating recorded)

2= Obstetrical estimate from 1st trimester ultrasound (maternal medical record: Expected due date (EDD) clinically assigned and 1st trimester US recorded)

3= Obstetrical estimate from 2nd trimester ultrasound with fetal biparietal diameter (BPD) dating within 2 weeks of sure last menstrual period (LMP); (maternal medical record: EDD clinically assigned, 2nd trimester ultrasound recorded, LMP recorded and certain)

4= Obstetrical estimate from 2nd trimester ultrasound with unsure or no LMP date; (maternal medical record: EDD clinically assigned, 2nd trimester ultrasound recorded, LMP uncertain or not recorded)

5= Best obstetrical consensus estimate; (maternal medical record: EDD clinically assigned with no ultrasounds documented during 1st and 2nd trimesters)

6=Obstetrical estimate from LMP only; (maternal medical record: EDD based only on LMP)

7=Neonatal estimate of GA at delivery; (childbirth medical record: Best estimate of completed weeks; assumed to be best obstetrical estimate)

8=Estimated from cohort research encounter during pregnancy; (maternal anthropometric form: Estimated GA at time of weight and height measurement during pregnancy)

9=Self-report by mother; (interview at birth: Number weeks pregnant at delivery, reported by biological mother)

10=Self-report by mother; (interview about total weight gain during pregnancy: Number weeks pregnant at delivery, reported by biological mother)

11= Cohort estimate of EDD; (participant registration into cohort: Administratively recorded EDD)

12= Report from caregiver or other respondent; (interview at birth: Number weeks pregnant at delivery, reported by someone other than the biological mother)
